# Effect of enhancing audit and feedback on uptake of childhood pneumonia treatment policy in hospitals that are part of a clinical network: a cluster randomized trial

**DOI:** 10.1186/s13012-019-0868-4

**Published:** 2019-03-04

**Authors:** Philip Ayieko, Grace Irimu, Morris Ogero, Paul Mwaniki, Lucas Malla, Thomas Julius, Mercy Chepkirui, George Mbevi, Jacquie Oliwa, Ambrose Agweyu, Samuel Akech, Fred Were, Mike English, Clinical Information Network Authors, Timothy Tuti, David Gathara, Susan Gachau, Samuel Ngarngar, Ivan Injira, David Kimutai, Caren Emadau, Cecilia Mutiso, Charles Nzioki, Agnes Mithamo, Margaret Kuria, Sam Otido, Grace Wachira, Peris Njiiri, Rachel Inginia, Melab Musabi, Sande Charo, Grace Ochieng, Lydia Thuranira

**Affiliations:** 1 Health Services Unit, Kenya Medical Research Institute/Wellcome Trust Research Programme, Nairobi, Kenya; 2 Department of Paediatrics and Child Health, University of Nairobi, Nairobi, Kenya; 3 Nuffield Department of Medicine, University of Oxford, Oxford, UK

## Abstract

**Background:**

The World Health Organization (WHO) revised its clinical guidelines for management of childhood pneumonia in 2013. Significant delays have occurred during previous introductions of new guidelines into routine clinical practice in low- and middle-income countries (LMIC). We therefore examined whether providing enhanced audit and feedback as opposed to routine standard feedback might accelerate adoption of the new pneumonia guidelines by clinical teams within hospitals in a low-income setting.

**Methods:**

In this parallel group cluster randomized controlled trial, 12 hospitals were assigned to either enhanced feedback (*n* = 6 hospitals) or standard feedback (*n* = 6 hospitals) using restricted randomization. The standard (network) intervention delivered in both trial arms included support to improve collection and quality of patient data, provision of mentorship and team management training for pediatricians, peer-to-peer networking (meetings and social media), and multimodal (print, electronic) bimonthly hospital specific feedback reports on multiple indicators of evidence guideline adherence. In addition to this network intervention, the enhanced feedback group received a monthly hospital-specific feedback sheet targeting pneumonia indicators presented in multiple formats (graphical and text) linked to explicit performance goals and action plans and specific email follow up from a network coordinator. At the start of the trial, all hospitals received a standardized training on the new guidelines and printed booklets containing pneumonia treatment protocols. The primary outcome was the proportion of children admitted with indrawing and/or fast-breathing pneumonia who were correctly classified using new guidelines and received correct antibiotic treatment (oral amoxicillin) in the first 24 h. The secondary outcome was the proportion of correctly classified and treated children for whom clinicians changed treatment from oral amoxicillin to injectable antibiotics.

**Results:**

The trial included 2299 childhood pneumonia admissions, 1087 within the hospitals randomized to enhanced feedback intervention, and 1212 to standard feedback. The proportion of children who were correctly classified and treated in the first 24 h during the entire 9-month period was 38.2% (393 out of 1030) and 38.4% (410 out of 1068) in the enhanced feedback and standard feedback groups, respectively (odds ratio 1.11; 95% confidence interval [CI] 0.37–3.34; *P* = 0.855). However, in exploratory analyses, there was evidence of an interaction between type of feedback and duration (in months) since commencement of intervention, suggesting a difference in adoption of pneumonia policy over time in the enhanced compared to standard feedback arm (OR = 1.25, 95% CI 1.14 to 1.36, *P* < 0.001).

**Conclusions:**

Enhanced feedback comprising increased frequency, clear messaging aligned with goal setting, and outreach from a coordinator did not lead to a significant overall effect on correct pneumonia classification and treatment during the 9-month trial. There appeared to be a significant effect of time (representing cumulative effect of feedback cycles) on adoption of the new policy in the enhanced feedback compared to standard feedback group. Future studies should plan for longer follow-up periods to confirm these findings.

**Trial registration:**

US National Institutes of Health—ClinicalTrials.gov identifier (NCT number) NCT02817971. Registered September 28, 2016—retrospectively registered.

**Electronic supplementary material:**

The online version of this article (10.1186/s13012-019-0868-4) contains supplementary material, which is available to authorized users.

## Introduction

Pneumonia is the second leading cause of child mortality worldwide; 0.9 million of the 5.9 million childhood deaths in 2015 were caused by pneumonia [1–3]. At least one half of all global pneumonia deaths occurred in sub-Saharan Africa [3]. The World Health Organization (WHO) pneumonia guidelines that diagnose pneumonia using a small number of simple clinical signs are routinely used in sub-Saharan Africa where resources for laboratory and radiologic diagnosis of pneumonia are limited [4]. Prior to 2013, WHO guidelines recognized three pneumonia severity classifications: pneumonia, severe pneumonia, and very severe pneumonia [5, 6]. According to previous guidelines, a child with a cough or difficult breathing was diagnosed with pneumonia if they developed fast breathing. Severe pneumonia was defined by lower chest wall indrawing in a child with cough or difficult breathing while a diagnosis of very severe pneumonia was made in children who developed any danger sign (inability to drink/breastfeed, altered consciousness, grunting, central cyanosis, hypoxia) in the presence of cough or difficult breathing. The severity of pneumonia guides subsequent antibiotic treatment. Emerging research, however, challenged this original guidance [5, 7]. First, studies suggested equivalence between injectable penicillin and oral amoxicillin for the treatment of pneumonia characterized by indrawing but no danger signs [8]. Second, studies suggested that the population of children with pneumonia and indrawing but no danger signs for whom recommendations suggested inpatient care could be successfully treated as outpatients [9].

The WHO responded to this new evidence in 2013 by making the first major revisions to childhood pneumonia guidelines in many years [4, 10]. These revisions aimed to (1) simplify the classification of childhood pneumonia severity to two categories instead of three and (2) switch treatment from injectable penicillin to oral amoxicillin for children aged 2–59 months with pneumonia characterized by indrawing but no danger signs (hereafter referred to as indrawing pneumonia). The treatment change harmonized the treatment for indrawing pneumonia with that for pneumonia defined only by fast breathing (without either indrawing or any danger signs). The 2015 results of a Kenyan trial also suggesting equivalence of injectable penicillin and oral amoxicillin (conducted on hospitalized patients) prompted a change in Kenyan pneumonia guidelines to align with the new WHO guidance in 2016 [11].

Progress in translating policy changes such as these for pneumonia into routine clinical practice is slow in sub-Saharan Africa [12], even in cases where clear guidance exists [13]. This slow progress is particularly evident for policies that require changes in well-established clinical behaviors [14]. For example, in Kenya, reducing the unnecessary use of cough medicines as co-treatment of pneumonia has taken many years [15], while changing first-line treatment for inpatient severe malaria has taken several years [16]. The advent of new pneumonia guidelines in Kenya therefore provided an opportunity to study implementation strategies that might accelerate adoption of the new guidelines.

Audit of hospital care is widely used as a strategy for improving quality of care. Clinical audit involves measuring clinical teams’ performance in providing care [17–19]. The effect of audit is enhanced when it is coupled with feedback where agents (e.g., peers or supervisors) provide information regarding individual health worker or clinical teams’ performance [17, 18]. The suggested mechanism through which audit and feedback improves performance in hospitals is by identifying and reducing the discrepancy between current and desired performance [20]. Interventions that make better use of audit and feedback provide a possible strategy for promoting desired clinical performance following changes in health policies. We therefore designed a study to compare the effect of alternative audit and feedback strategies delivered concurrently with dissemination of the new policy on the uptake of policy recommendations. Our trial aimed to test whether enhancing the frequency and format of feedback and incorporating goal setting and action planning could result in more rapid adoption of the new policy of using oral amoxicillin for indrawing pneumonia. This randomized trial directly addresses a need expressed in a systematic review that included 140 studies that “future studies of audit and feedback should directly compare different ways of providing feedback” [21]. Addressing the question of how best to promote policy adoption is also important as failure to implement evidence-based policies in routine practice undermines the whole research enterprise.

## Methods

### Study design

A pragmatic parallel group cluster randomized trial was conducted over a 9-month period in 12 county hospitals providing first level inpatient referral care in Kenya that are part of a clinical information network (CIN) [22, 23]. The hospital was the unit of randomization. The cluster design was selected because inpatient pediatric care is organized around multidisciplinary teams composed of doctors and nurses among other health workers and providing team level feedback is logistically easier for health systems. Secondly, it is much more effective to deliver the intervention at hospital level rather than individual health worker level in hospitals with rapid staff turnover where the individual, junior front-line clinicians change every 3 months. This is the group of clinicians who are expected to adhere to the pneumonia guidelines.

### Study participants

The 14 hospitals in CIN were selected in consultation with the Ministry of Health from 12 out of the 47 counties in Kenya. All these hospitals were public government-owned hospitals and were estimated to admit at least 1000 children per year and located in one of the main malaria ecological zones in Kenya (either high or low to very low malaria transmission). For the trial, 12 of the initial 14 hospitals, one from each county, were selected for inclusion. The hospitals that were excluded from the trial were a small facility (less than 1000 admissions per year) that is staffed by clinical officers (physician assistants) and not resident physicians or pediatricians as in the other 13 CIN hospitals. The second facility was excluded from the trial because it had existing formal linkages with other research and academic institutions that might influence the intervention effect.

Individual case records were identified for post-discharge medical record review in each participating hospital if the admissions were aged between 2 and 59 months and had a provider clinical diagnosis of pneumonia regardless of signs or symptoms. We excluded admissions with cough persisting more than 2 weeks or co-morbid conditions for which there are specifically recommended antibiotic treatments in Kenyan guidelines including meningitis, HIV, severe malnutrition, severe malaria (spanning cerebral malaria and severe malaria anemia), surgical conditions, and sepsis [24].

### Interventions

The trial had two arms, and each trial arm received one of the two intervention strategies implemented between March 10, 2016, and December 4, 2016. The first intervention (referred to as enhanced feedback) involved an audit on indicators of pneumonia care with feedback delivered every month, using a specific pneumonia feedback sheet provided in both graphical and text-based formats. We incorporated explicit performance goals for each audit and feedback cycle with accompanying action plans for relevant indicators. The network coordinator (a senior pediatrician) sent emails to the local pediatrician encouraging them to read and act on the received feedback. The enhanced feedback intervention was designed drawing on Feedback Intervention Theory [20], the modifications to this theory suggested by Hysong and colleagues [18, 19], and a systematic review that suggests which attributes of feedback may promote its effectiveness [21].

The second intervention (referred to as standard feedback) was audit and feedback of general pediatric care delivered less frequently (bimonthly), using a single format of comprehensive performance reports for general indicators of pediatric care spanning five major conditions including pneumonia and described in more detail elsewhere [25]. Additionally, both trial arms received a half-day training delivered to the clinical and nursing teams on the new pneumonia guidelines at the start of the trial. Clinicians in all hospitals were also supplied with updated protocol booklets that contained information on the new pneumonia guidance including specific pneumonia algorithms articulating the key clinical signs and how these are to be used in classification together with dosage tables for oral amoxicillin. Finally, all hospitals in the trial received continued network support, described in detail in the next section under intervention context.

#### Intervention context

The Kenyan clinical information network has been in operation since late 2013 and has adopted a range of approaches to understand and improve hospital care (Table 1). A full description of its components, activities, and theoretical underpinnings is presented elsewhere [26]. In brief, CIN components and activities comprise (1) initial mentorship and basic training on team management provided to hospitals’ pediatricians in 2014 and early 2015 to promote their engagement in service improvement, (2) data collection that supports the provision of hospital-specific reports on quality of documentation and adherence to multiple guidelines (spanning recording of key clinical signs, appropriate use of basic diagnostic tests and assessment and treatment of malaria, pneumonia, diarrhea & dehydration, meningitis, and severe acute malnutrition), and (3) peer to peer networking through twice yearly meetings and a simple WhatsApp group. Hospital reports are sent as printed documents and by email to the pediatrician, the lead pediatric nurse and the medical superintendent in each hospital; the pediatrician is expected to provide feedback to frontline pediatric care providers on their hospital’s performance. Prior to the trial, over the period 2014 to 2015, all hospitals had received nine comprehensive performance reports. During the trial period, all 12 hospitals continued to receive wider network support (captured in items b and c above) including the comprehensive feedback reports sent every 2 months. From the start of the trial these standard reports contained, among much other information, feedback in the form of tables of indicators of hospitals’ correct performance in documentation of pneumonia clinical signs, pneumonia classification, and pneumonia treatment reflecting adherence to the new guidelines.

**Table 1:** Key components of the multifaceted intervention to improve adoption of new pneumonia policy

Intervention	Description	Treatment arm involved
Network strategy	o Sensitization of hospital pediatric care teams on quality healthcare service by coordinating team o Initiative to improve quality of pediatric data through providing minimal ICT infrastructure (desktop computer), introduction of standardized pediatric admission record form, and recruitment of data clerk at each hospital o Establishment of clinical information network bringing together Ministry of Health, pediatric professional organization, hospital team leaders, and senior peers from a local University and a research organization o External facilitation of quality improvement efforts by network coordinating team through 6 monthly face-to-face network meetings and communication with pediatricians (via phone, email, social media) and a total of 2 or 3 hospital visits in 2014 and early 2015	Both intervention and control
Standard audit and feedback	o Developing and implementing tools for monitoring indicators of quality of general pediatric care o Two to three-monthly feedback reports of performance assessed against national guidelines for pediatric care o Each hospital’s performance compared to its own performance in the preceding period and also compared anonymously to other network hospitals o General encouragement offered to improve all aspects of care by coordinator to team leaders by 2-monthly phone/email communication	Both intervention and control
Enhanced audit and feedback	o Definition of goals for adoption of new pneumonia* policy o Monthly feedback in text of: ▪Hospital performance in comparison with goals for indicators of correct pneumonia classification and treatment as contained in new policy ▪Hospital performance in comparison with anonymized performance information of other hospitals o Graphical representation of each hospital’s performance trend by month from the start of the intervention o Specific follow up on pneumonia indicator performance by coordinator with team leaders by monthly phone/email communication	Intervention only

*Specific goals included attaining at least 80% compliance with classification and treatment for childhood pneumonia admissions

#### Revised pneumonia policy

WHO and Kenyan guidelines use simple clinical signs to identify children with pneumonia and then assign a classification for severity of pneumonia in settings with little or no access to diagnostic technology. The new guidelines recommend which among three possible case management actions is applicable (after appropriate treatment for wheeze where indicated): (1) symptomatic treatment for a simple upper respiratory tract infection without giving antibiotics, (2) treatment with oral Amoxicillin for fast breathing or indrawing but no danger signs, (3) providing supportive care and combination injectable antibiotic treatment where there are danger signs.

This trial targeted the second case management action, i.e., treatment with oral Amoxicillin for fast breathing or indrawing but no danger signs. The dispersible formulation of oral Amoxicillin was supplied to all hospitals during the entire trial period (courtesy of UNICEF Kenya). This continuous and reliable supply ensured that differences in drug supplies did not impact on implementation of the intervention or the resulting intervention effect.

### Outcomes

The primary outcome was the proportion of all admitted patients with pneumonia characterized by fast breathing or chest indrawing who were both correctly classified and treated in the first 24 h. For this outcome, prescription of correct treatment (i.e., oral amoxicillin for pneumonia) was contingent on correct classification of pneumonia severity. Based on the clinical signs documented in the medical record during admission, we determined whether a child had pneumonia defined by chest indrawing or fast breathing (> 50 breaths per minute in neonates and > 40 breaths per minute in children aged 1 to 59 months). We then checked the records to establish which of the two alternative classifications—pneumonia or severe pneumonia—recommended in the new guidelines was assigned by the clinician. Children correctly classified as (indrawing) pneumonia and with clinical signs consistent with this classification who were also prescribed oral amoxicillin were regarded as meeting the primary outcome definition. Those who had incorrect classification (severe pneumonia according to new guidelines or other classification not contained in new guidelines) were considered not to have met the primary outcome definition irrespective of whether they were prescribed oral amoxicillin. Similarly, children with correctly classified (indrawing) pneumonia who were not prescribed oral amoxicillin did not meet the primary outcome definition.

We also explored a secondary descriptive outcome, the proportion of correctly classified and treated children with (indrawing) pneumonia for whom clinicians changed treatment from oral amoxicillin to injectable antibiotics. The new guidelines recommend a switch to injectable antibiotics in the following cases: first, at any time when a child progresses from (indrawing) pneumonia to severe pneumonia during the course of oral treatment; second, at 48 h in case (indrawing) pneumonia does show improvement in at least one sign (respiratory rate, severity of indrawing, fever or ability to drink); and third, during day 5 of treatment if at least three of the following criteria are present: fever; respiratory rate above 60 per minute; cyanosis; persistent chest indrawing or worsening chest x-ray.

### Sample size

We estimated that at least 680 patients with (indrawing) pneumonia would be required per arm to evaluate the primary outcome and that such numbers would be achievable in a period of 9 months after trial commencement with six hospitals per arm. We assumed an average cluster size of 113 patients and an ICC of 0.2 (corresponding to a VIF of 15.8 derived from patterns of pneumonia classification and treatment in CIN hospitals prior to trial commencement) and Type I error of 5%. This provided 90% power to detect a two-fold increase in the odds of correct (indrawing) pneumonia classification and treatment assuming a 30% compliance with the guideline-recommended pneumonia classification and treatment in the standard feedback group.

### Randomization

The trial statistician performed restricted randomization of 12 hospitals that involved randomly selecting a single allocation of clusters to treatment arms from a subset of all the possible allocations of six hospitals per arm that retained balance on key covariates. We aimed to ensure relative balance was achieved between treatment arms in terms of geographic location as a proxy for malaria prevalence (2:3 or 3:2 split of the five hospitals located in western Kenya) and monthly pneumonia admissions (3:4 or 4:3 split of the seven hospitals admitting < 30 pneumonia admissions per month). Details of the randomization have been published [27], but in brief there were 924 possible allocations of 12 clusters into two groups of which 536 allocations met the defined balancing criteria, and one of these 536 allocations was randomly selected by the statistician using computer-generated random numbers (R software 3.3.3) [28].

### Data collection

The data collection and management procedures have been described in detail elsewhere [23, 29]. In brief, data were entered into an electronic database from inpatient paper-based medical records at the point of discharge. A data clerk at each hospital was trained on abstracting clinical data from medical records using standard operating procedures. These clerks who were blinded to group allocation documented patient biodata, admission clinical features and history of illness, initial and subsequent antibiotic treatment, and discharge outcomes. The clerk synchronized data to a centralized server hosted at the KEMRI-Wellcome Research Programme on a daily basis after running error checking scripts and reviewing any entries that had queries (out of range values, plausibility checks). A second round of error checking was done after synchronization in the main servers and any further queries communicated to the clerks by telephone and resolved where possible. Lastly, three monthly external data quality assurance (DQA) were conducted by a research team that visited the hospital and reentered a random selection of approximately 10% of observations entered in the database during the period preceding the DQA [27].

### Data analysis

The study protocol containing the pre-specified analysis plan has been published [27]. For the analysis of the primary outcome, we have fitted a hierarchical logistic regression model. The dependent variable was an indicator of whether or not the child admitted with (indrawing) pneumonia was correctly classified and treated according to new guidelines (coded 1 for children who were correctly classified and treated, otherwise the indicator was coded 0). We hypothesized that a gradual and cumulative change might occur in adherence to the new guideline with each successive feedback cycle. We therefore included independent variables representing trial arm (standard versus enhanced feedback), time of patient admission to hospital in relation to duration of intervention (a variable indicating time in completed months—between 1 and 9—calculated from the start of the study to the admission date), and an interaction between trial arm and time (trial arm × time). The interaction term was used to estimate the difference in the gradient of slopes for intervention and control arms. Therefore, term represented the difference in the change in primary outcome over the 9-month follow-up between the two groups [30]. We tested the assumption that for both groups, the log odds of correct treatment and classification in the logistic model were a linear function of time through modeling nonlinear relationships. We used random intercept models to account for variation in performance between hospitals in the first follow-up month before any feedback had occurred. To adjust for the small number of clusters (*n* = 12) and large variation of cluster sizes that are associated with an inflated Type I error, we estimated mixed logistic models using adaptive Gaussian-Hermite approximations to the likelihood. This approach yields less biased estimates with few clusters compared to Laplace approximation or penalized quasi-likelihood [31–33]. Bootstrapping was used to obtain the reported confidence intervals. Adjustment for case mix between hospitals was done by including patient-level factors in the model, namely sex and age. At the hospital level, we included indicator variables to show whether a hospital was located in a malaria-endemic area, and a separate variable indicating whether it was in a rural or urban setting. We performed a descriptive analysis of the secondary outcome of antibiotic switch from oral to injectable antibiotics to explore clinically suspected treatment failure as a measure of perceived safety of oral amoxicillin. This study is reported as per the Consolidated Standards of Reporting Trials (CONSORT) for cluster trials guidelines (Additional file 1: Table S1).

#### Multiple imputation

During exploratory analysis, we established that the proportion of missing data for individual clinical sign variables relevant for classifying pneumonia severity was between 2 and 15%. We therefore used multiple imputations to handle missing data and allow classification of all pneumonia patients. We used Bayesian bootstrap predictive mean matching to impute missing data 50 times. The imputed variables included numeric covariates (e.g., age) and categorical variables (clinical signs). After imputation, diagnostic checks were conducted to compare distribution of imputed and observed data. Each imputed dataset was analyzed and the estimates combined using Rubin’s rules [34]. Sensitivity analyses were conducted to assess robustness of inference to possible departure from missing at random assumptions.

## Results

### Delivery of the enhanced feedback intervention

**Fig. 1:**
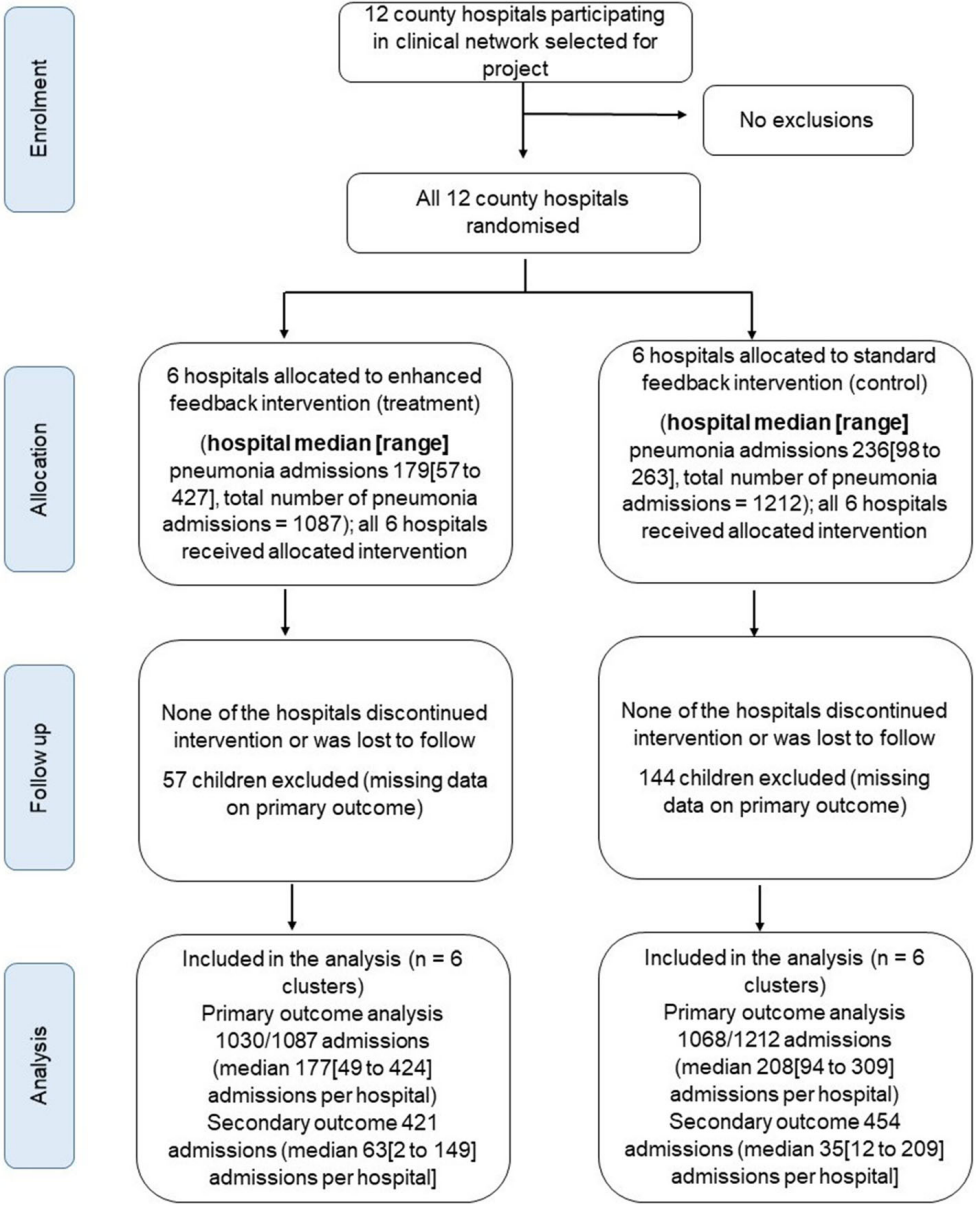
Flow diagram of progress of clusters and individuals through the cluster RCT

The feedback intervention was successfully delivered to all the 12 hospitals that had been randomly assigned to enhanced or standard intervention (Fig. 1). All hospitals followed the planned timing and frequency of feedback provision. Nine months after the start of the study, eight rounds of monthly pneumonia-specific feedback had been delivered in each intervention hospital and four rounds of bimonthly general feedback had been provided to hospitals in both trial arms. Enhanced feedback reports were dispatched on the same day to all intervention hospitals throughout the study period as per the original plan, and general feedback reports were sent out to all hospitals within 2 days of the date on which they were due. None of the hospitals withdrew from the study.

### Trial participants

There were a total of 2299 eligible (indrawing) pneumonia admissions to all 12 participating hospitals during the trial period. Of these patients, 1087 were from hospitals assigned to enhanced feedback and 1212 from hospitals assigned to standard feedback. The median number of pneumonia admissions to hospitals in the enhanced feedback trial arm was 166 (range 47 to 383) and that of admissions to the standard feedback arm was 202 (range 89 to 329). Comorbid illnesses were common (66.5%) in admissions even after exclusion of patients with HIV, severe malnutrition, meningitis, and severe malaria for whom different antibiotic regimens are recommended (Table 2).

**Table 2:** Characteristics of hospitals and patients participating in the CIN audit and feedback intervention trial

Characteristics	Enhanced feedback	Standard feedback	Total
Cluster level			
No. of hospitals, *n*/*N*	6/6	6/6	12/12
No. of hospitals with > 1 pediatrician*, *n*/*N*	2/6	1/6	3/12
No. of hospitals in malaria-endemic regions, *n*/*N*	3/6	2/6	5/12
No. of hospitals with > 40 pediatric beds, *n*/*N*	2/6	2/6	4/12
Individual level			
Pneumonia admissions	1087	1212	2299
Median age in months, (IQR)	12 (7–17)	14 (8–18)	13 (8–24)
Males, *n*/*N* (%)	604/1086 (55.6%)	661/1200 (55.1%)	1265/2286 (55.3%)
Duration of illness in days, median (IQR)	3 (2–5)	3 (2–5)	3 (2–5)
Referred to hospital, *n*/*N* (%)	101/860 (11.7%)	179/724 (17.4%)	280/1884 (14.9%)
Clinical findings during admission documented using a structured admission record form**, *n*/*N* (%)	1069/1083 (98.7%)	1199/1211 (99%)	2268/2294 (98.9%)
Wheezing present during admission, *n*/*N* (%)	83/1084 (7.7%)	112/1203 (9.3%)	195/2287(8.5%)
Comorbid diagnosis present at admission***, *n*/*N* (%)	692/1027 (63.7%)	827/1212 (68.2%)	1519/2299(66.1%)
Mid-upper-arm-circumference, median (IQR)	14 (13–15)	14 (13–15)	14(13–15)
Length of hospital stay, median (IQR)	2 (1–5)	3 (2–6)	3 (1–5)
Died, *n*/*N* (%)	22/1087 (2.0%)	37/1212 (3.1%)	59/2299 (2.6%)

*All remaining hospitals had a single pediatrician

**Structured admission forms were introduced within network hospitals to improve quality of documentation of features of illness, investigations and management

***Any comorbid illness not excluded in trial exclusion criteria, e.g., dehydration

The baseline characteristics of hospitals and pneumonia admissions to the hospitals were similar between trial arms (Table 2). The median age of children admitted with pneumonia was 13 months (interquartile range 8–24 months). One hundred and ninety-five out of all pneumonia admissions (8.5%) had a wheeze during admission which is a guideline criterion for considering a possible diagnosis of asthma. Treatment with bronchodilators is recommended in children with wheeze and the initial bronchodilator treatment could lead to a revision of pneumonia classification. Out of the admissions with information on whether they had received prereferral treatment at a separate health facility prior to admission in hospitals participating in the study, 14.9% (280 out of 1884) had documentation showing that they were referred from other health facilities to the admitting hospital. Discharge outcome following hospitalization for (indrawing) pneumonia treatment was also similar across the two trial groups. The median length of stay was 3 days (IQR 1–5 days). The overall mortality among pneumonia admissions was 2.6% (59 out of 2299) with group-specific mortality of 2.0% (22 out of 1087) and 3.1% (37 out of 1212) in the enhanced and standard feedback groups, respectively.

### Primary outcome

We assessed a contingent primary outcome in which correct treatment was determined in the group of children who had correct (indrawing) pneumonia classification. During the 9-month trial period, 615 out of 1087 (56.6%) and 742 out of 1212 (61.2%) pneumonia admissions were correctly classified in the enhanced and standard feedback groups, respectively. Data on antibiotic prescriptions within 24 h of admission were not available for 57 (5.2%) and 144 (11.9%) children in the enhanced and standard feedback trial arms, respectively (Fig. 1). Therefore, the primary outcome of correct pneumonia classification and treatment could not be determined in these admissions without antibiotic prescriptions resulting in the assessment of primary outcome in 1030 children in the enhanced feedback group and 1068 children in the standard feedback group. For the primary outcome, 393 out of 1030 children in the enhanced feedback group (38.2%) and 410 out of 1068 children in the standard feedback group (38.4%) were correctly classified and treated using oral amoxicillin during the entire 9-month period (odds ratio 1.11, 95% CI 0.37–3.34; *P* = 0.855). The children with the primary outcome represented 63.9% (393/615) and 55.3% (410/742) of correctly classified children in enhanced and standard feedback groups, respectively.

**Fig. 2:**
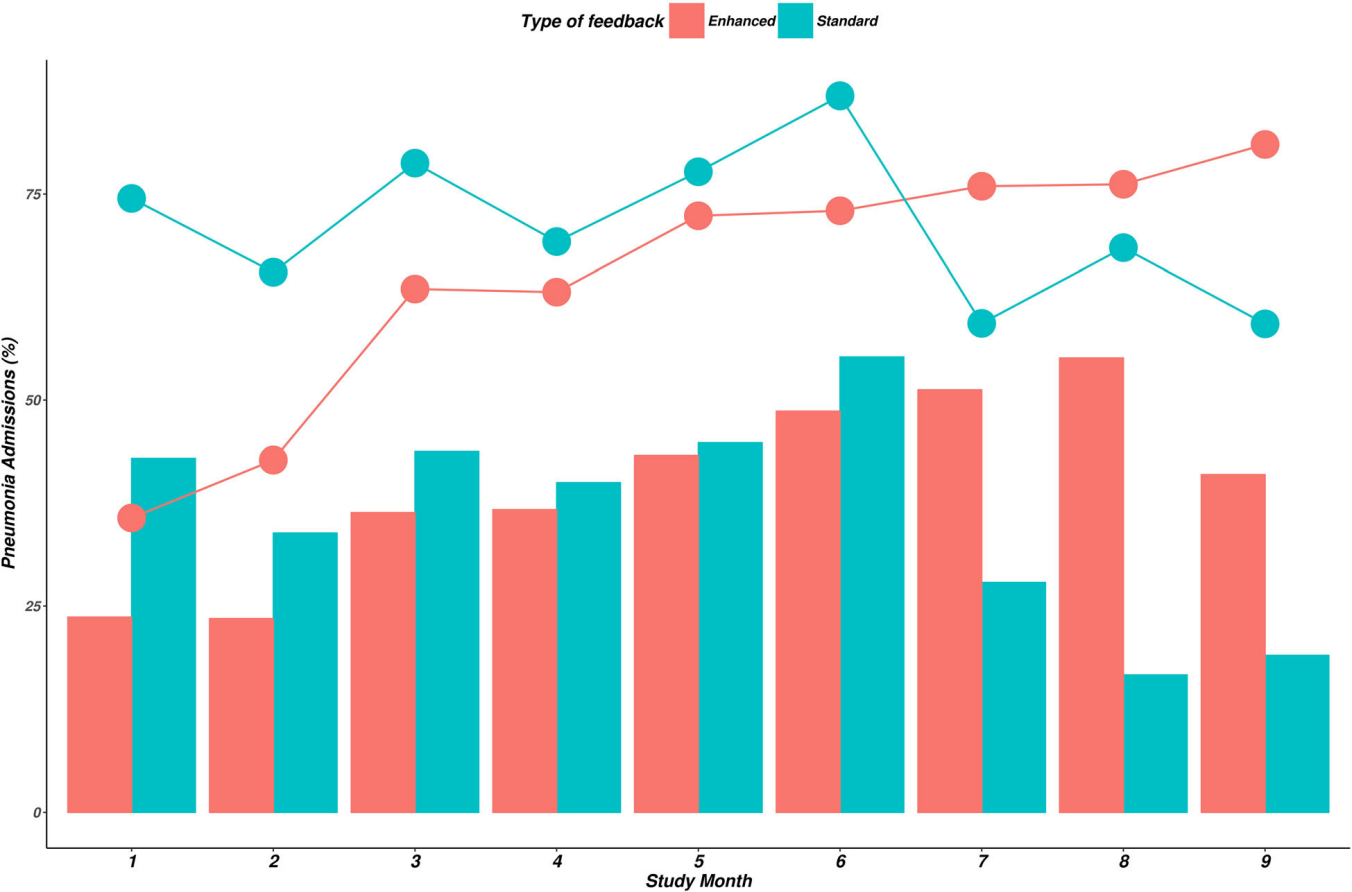
Correct classification and treatment of childhood pneumonia admissions according to duration of intervention and type of feedback intervention

The enhanced feedback hospitals had poorer classification and treatment practices at baseline OR 0.54, 95% CI 0.19 to 1.68 (Table 3). On exploring monthly performance of correct pneumonia classification and treatment after each round of enhanced feedback, there was an improvement in the enhanced feedback group while performance declined in the standard feedback arm (Fig. 2). The decline after month 7 in standard feedback arm was attributable to consistently poor performance in four out of the six facilities (Additional file 2: Figure S1). The coefficient for the interaction term between intervention group and time (in months) provided evidence of a significant difference in the slope of change with time between the trial arms OR = 1.25, 95% CI 1.14 to 1.36 (Table 3). The odds of correct practices increased by 19% per month in the intervention arm while the estimated odds declined by 5% per month in the control group.

**Table 3:** Effect of audit and feedback intervention on correct classification and treatment of childhood pneumonia admissions in CIN hospitals

	Primary model estimates		Multiple imputation model (sensitivity analysis)
	Adjusted OR (95% CI)	*P* value	Adjusted OR (95% CI)
Enhanced audit and feedback intervention	0.54 (0.19, 1.68)	0.270	0.52 (0.18, 1.53)
Time (in months)	0.95 (0.89, 1.01)	0.090	0.95 (0.90, 1.01)
Time × feedback intervention	1.25 (1.14, 1.36)	< 0.001	1.24 (1.14, 1.35)
Age in months	1.00 (0.99, 1.01)	0.749	1.00 (0.99, 1.01)
Male	1.07 (0.87, 1.35)	0.496	1.06 (0.87, 1.29)
Malaria-endemic area	0.6 (0.22, 1.4)	0.338	0.61 (0.22, 1.65)
Hospital located in urban area	0.41 (0.14, 1.32)	0.142	0.42 (0.13, 1.28)

**Fig. 3:**
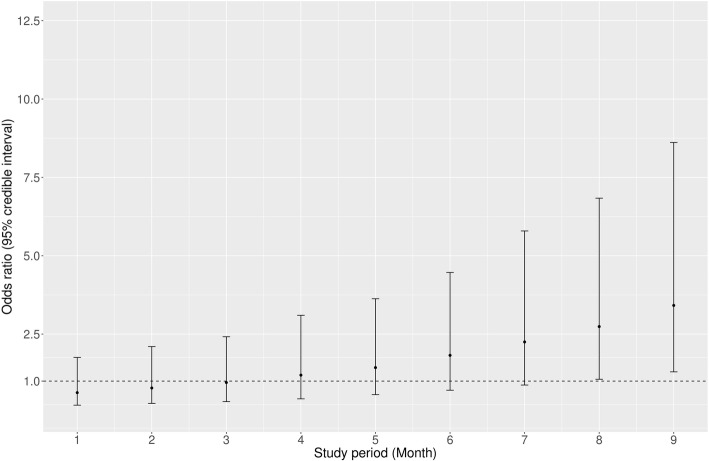
Odds ratios for correctly classifying and treating pneumonia in enhanced compared to standard feedback hospitals. At each time point (months 1 to 9), we estimated the odds of correct classification and treatment for patients admitted during the immediately preceding one-month period that coincided with dissemination of the monthly enhanced feedback reports in the intervention arm. The odds ratios (95% CI) are then plotted at these monthly time points and show the predicted odds of correct classification and treatment of pneumonia admissions in the enhanced feedback trial arm compared to the standard feedback arm (adjusted for patient and hospital level factors)

The predicted odds of correct classification and treatment of (indrawing) pneumonia admissions did not differ significantly between the trial arms during the initial months of the trial (Fig. 3). However, performance in classifying and treating childhood pneumonia was better in the enhanced feedback arm by the ninth month (OR 3.6, 95% CI 1.17 to 11.1).

The sensitivity analysis conducted on multiply-imputed data is presented in Table 3. Findings of the analysis were consistent with those of the primary analysis (Additional file 3).

### Secondary outcome

There were 91 patients who had their antibiotics switched from oral to injectable antibiotics among the correctly classified and treated children yielding an overall prevalence of 12.7% (95% CI 10.4 to 15.4). Forty-three out of 340 (12.6%, 95% CI 9 to 16.7) patients in the enhanced feedback group and 48 out of 375 (12.8%, 95% CI 9.6 to 16.6) patients in the standard feedback group who were initially correctly classified and treated with oral amoxicillin had a treatment switch to injectable antibiotics.

## Discussion

We assessed whether providing enhanced feedback to clinical teams is effective in improving the uptake of new guidelines for treatment of (indrawing) pneumonia in hospitals. In this trial, we did not show an overall difference in correct classification and treatment of childhood pneumonia. Approximately 40% of pneumonia admissions in each of the two trial arms were correctly managed according to the new guidelines over the entire 9-month trial period. In literature, the evidence for the effect of multifaceted practice change interventions remains mixed [35, 36]; although, we have previously shown benefits in the Kenyan hospital setting [37]. Observational analyses conducted in participating hospitals prior to the onset of this trial also show the network intervention has improved clinical documentation [29], for example, ascertainment of HIV status and adoption of MUAC measurement [38].

The standard feedback provided within the network is hospital-specific with peer comparison data, is provided regularly, using multiple modes of delivery (paper, electronic, text and graphical presentation), but includes pneumonia policy adherence indicators within a comprehensive performance report spanning multiple common conditions [27]. This standard feedback had been provided for over 2 years to each hospital prior to the trial. It is accompanied by network activities aimed at promoting clinical leadership through mentorship linked to twice yearly face-to-face network meetings, peer to peer support through a WhatsApp group, and expert outreach by a senior clinical coordinator [17, 26].

The enhancements to standard feedback we tested were use of a specific feedback form, increased frequency of feedback, use of goal setting for target indicators linked to suggested actions for improvement, and follow-up of feedback by a senior pediatrician. Such enhancements were selected on the basis of theory and inferences drawn from prior systematic reviews on aspects of feedback that show most promise [17, 18, 20]. We hypothesized that these elements when combined would improve the self-efficacy and motivation to promote policy adoption among the lead pediatricians particularly and their junior team members who actually provide admission care.

The trial, conducted in a resource-constrained healthcare setting, showed that the overall adoption of the new policy after initial training, guideline dissemination, and in the context of the network activities and feedback approaches we have described was moderate at approximately 40%. Failure to use recorded signs to direct treatment (misclassification) rather than inappropriate treatment once correctly classified accounted for most cases of incorrect performance. Initial training and dissemination of guidelines appeared to result in modest (and variable) adoption of the new policy. Subsequently, there was declining performance in hospitals receiving standard feedback (from a higher baseline) and increasing performance with enhanced feedback. This is consistent with the possibility that clinicians in the standard feedback arm slowly revert to prior behavior [39], whereas we speculate that enhanced feedback aimed particularly at the clinical team leader sustained and promoted guideline adoption among junior team members possibly through better reinforcement practices.

The decline in performance in the control arm may also be linked to staff rotations that occur frequently in Kenyan hospitals. Frontline staff who admit patients to district hospitals are typically intern clinicians in their first year of experiential training in countries like Kenya. Such staff typically rotate every 3 months and two such rotations occurred during this trial across both trial arms. The decline in performance in standard feedback hospitals may therefore have resulted in care increasingly being provided by clinicians who had not had specific training on the guidelines. Efforts were made to make sure these new clinicians had copies of guideline booklets. In the enhanced feedback hospitals, we suggest the pediatricians leading practice made a greater effort to support guideline adoption providing some insulation against staff turnover; although, we were unable to collect data on the actual practices of pediatric team leaders in intervention and control hospitals that might support this proposition. However, this suggestion is consistent with previous work in similar settings in which we observed that new practices became slowly embedded in hospital routines with long-term feedback despite considerable staff turnover [37].

The enhanced feedback intervention may have achieved more sustained adoption of the policy compared to standard feedback, but our findings suggest that there is still considerable scope for improving policy adoption requiring additional interventions. For example, neither the enhanced nor the standard feedback arm achieved the target performance of 80% compliance with correct classification and treatment for pneumonia. The failure to attain this goal could be explained by the level of goal difficulty. Greater success was achieved with classification of pneumonia severity using simple clinical signs, but performance declined when we examined the composite outcome with an additional requirement of prescribing appropriate treatment for the assigned pneumonia classification. Thus, the overall performance for correct classification in the trial ranged between 64% and 74% in the trial arms, but up to 32% of children in the control sites did not receive oral amoxicillin despite having been correctly classified (indrawing) pneumonia. There remains a need to investigate why clinicians have trouble adhering to treatment guidelines once they have correctly classified a child. A possible explanation is a lack of belief among clinicians in oral treatment or monotherapy hence the tendency to assign a more severe pneumonia classification to warrant use of injectable or broader spectrum antibiotics [40]. A useful additional finding from our study was that treatment failure among children given oral amoxicillin was similar to that of children who were started on injectable penicillin, a finding supporting the results of previous randomized trials [9, 11]. Demonstrating that almost nine out of ten children quickly improve on oral treatment may help provide some assurance to clinicians that oral treatment for (indrawing) pneumonia confers no more risk than injectable treatment, knowledge that may facilitate wider policy adoption. However, non-trivial mortality in this group of children in hospital does suggest the need for additional research factors that would help clinicians assess risk and additional on interventions to improve outcomes.

The optimal duration of time for providing feedback interventions to promote policy adoption is unclear [21], and it is possible that greater improvements would have occurred if the intervention was implemented for a longer period. This is suggested by the apparent benefits of enhanced feedback seen in the final months of the intervention period. There is a need for studies to determine how long it would take to embed new policies in practice. Future feedback intervention designs could also target individual clinicians as an additional approach to promote policy uptake especially during health provider rotations that occurred frequently in our trial. This could be done using phone text messaging which had been implemented successfully in previous randomized trials [41]; although, logistically, such interventions would be much harder to sustain at scale.

At present there is still limited work on how to implement new national policies at scale in LMIC. The failure to monitor implementation means much research evidence is not effectively translating to health benefits. We have shown that modest investments in information systems may support improvements [16, 23, 25, 29], and could support audit and feedback interventions. Investments in information systems within the field of adult and pediatric HIV care have shown that it is feasible to use such systems to improve antiretroviral therapy guideline adherence at scale under routine care conditions in LMICs [42, 43]. We argue that greater attention needs to be paid to improving information systems not just aimed at centralized national reporting but to enable monitoring and develop feedback interventions that promote adoption of effective interventions as part of wider efforts to reduce newborn and child mortality [44].

### Strengths

The cluster RCT design and methodologic rigor applied in the evaluation are a strength. Although we could randomize only 12 hospitals, we performed restricted randomization to minimize baseline imbalance in characteristics between trial arms (a major challenge in cluster trials). We also used random intercept multilevel models to account for any residual imbalances in performance at baseline therefore improving our ability to attribute any effects to intervention. To our knowledge, this is among the first studies conducted in low- and middle-income African countries to examine different approaches to providing feedback at hospital level while exploring characteristics of feedback that can optimize effectiveness. Our study was based on a theory-driven intervention that responded to current priority research areas within this field [17] and was conducted within a network with good quality data.

### Limitations

There are several limitations of the current study. First, neither the trial investigators nor the participants were blinded. However, the clerks who were responsible for data collection were unaware of the hospital allocation to intervention reducing the potential of differential documentation of physical examination findings, pneumonia severity classification, and treatment during data abstraction. Separately, contamination of intervention could have occurred through peer to peer interactions during the single face-to-face network meeting held while this trial was in progress and possibly through discussions on a social media platform (WhatsApp). We did not determine the extent of contamination but the decline of performance in the control arm suggests that this potential contamination was minimal and did not strongly influence the intervention effect. Separately, we did not study a random sample of hospitals and the findings in this study are most directly generalizable to county hospitals that provide primary referral inpatient care and that are also in receipt of some form of routine performance feedback (a situation that is rare in LMIC). Despite this limitation, we believe these findings are relevant to LMIC hospitals particularly as electronic medical records (EMR) are rapidly being introduced in many countries. Our findings further suggest that the rollout of EMRs should be accompanied by careful planning to incorporate appropriate data collection and enable further testing of audit and feedback. A further limitation of this trial is that due to reliance on information obtained from medical records at discharge, it is difficult to determine whether the intervention improved the actual care received, documented practice, or both.

## Conclusions

We have demonstrated that enhanced feedback as opposed to standard feedback delivered as components of a multifaceted network intervention did not result in improvements in the overall proportion of children managed in accordance with the new guidelines. However, providing enhanced feedback was associated with improving compliance over time. Findings suggest specific components of feedback including its frequency, links between goal setting and targeted action plans, and reinforcement by a senior professional may promote slow adoption of policy recommendations. Overall, policy makers, researchers, and implementers should pay more attention to utilizing and testing evidence-based strategies including audit and enhanced feedback to achieve policy adoption in low and middle income countries if the benefits of research investments and new interventions are to be realized.

## Additional files

Additional file 1: CONSORT checklist for reporting of cluster randomised trial. (DOCX 29 kb)

Additional file 2: Pneumonia severity classification during intervention period according to trial arm. (PPTX 47 kb)

Additional file 3: Example of hospital-specific pneumonia feedback sheet provided to the enhanced feedback arm. (DOCX 87 kb)
