# Supplementary figures and images for: Effect of enhancing audit and feedback on uptake of childhood pneumonia treatment policy in hospitals that are part of a clinical network: a cluster randomized trial
Source: Implement Sci. 2019 Mar 4;14:20. doi: 10.1186/s13012-019-0868-4 (PMC6398235; doi:10.1186/s13012-019-0868-4)

## Slide 1
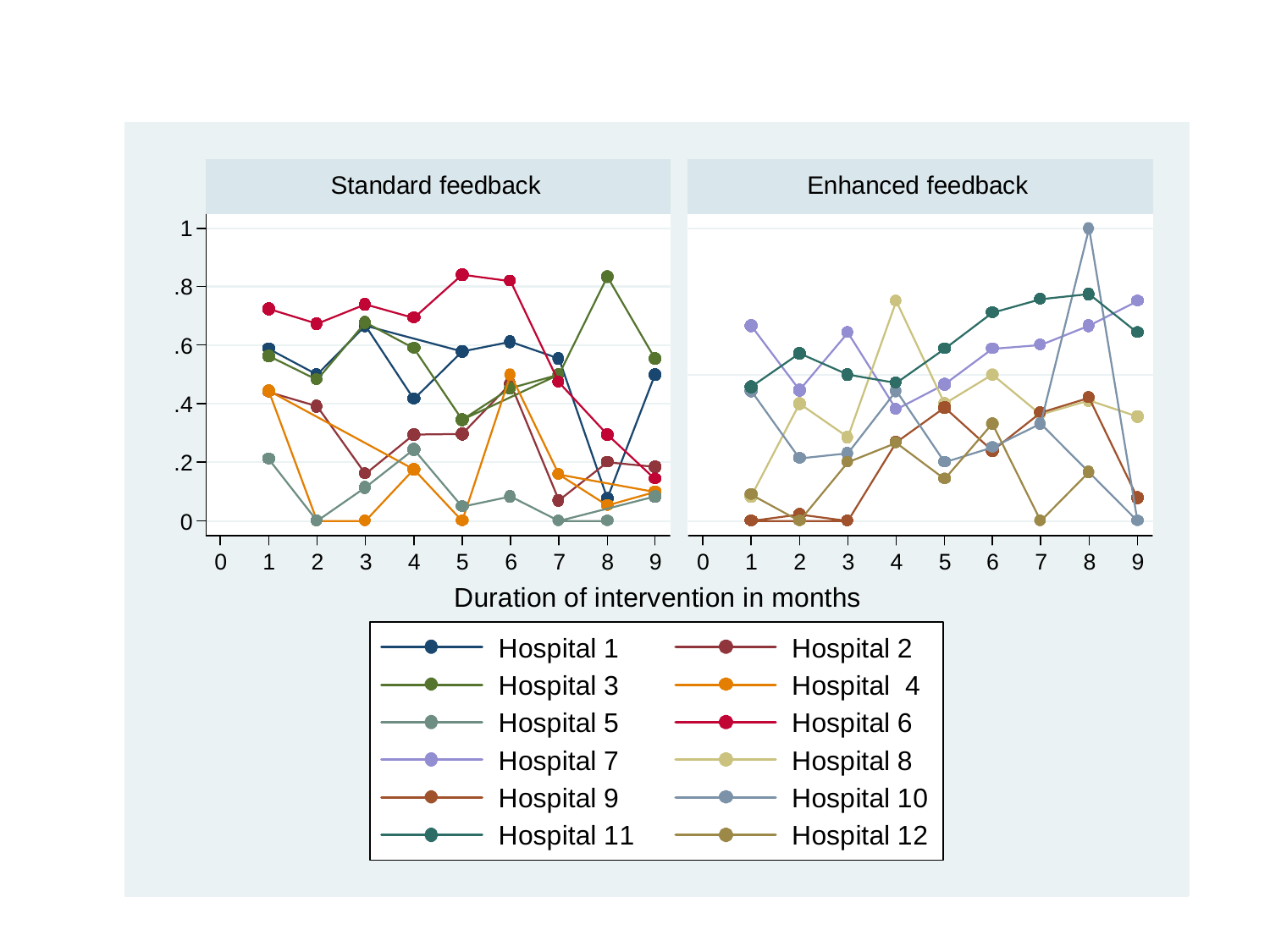

Supplement: Supplementary file 2 — Pneumonia severity classification during intervention period according to trial arm. (PPTX 47 kb) [file 13012_2019_868_MOESM2_ESM.pptx]
