# Supplementary material for: Effect of enhancing audit and feedback on uptake of childhood pneumonia treatment policy in hospitals that are part of a clinical network: a cluster randomized trial
Source: Implement Sci. 2019 Mar 4;14:20. doi: 10.1186/s13012-019-0868-4 (PMC6398235; doi:10.1186/s13012-019-0868-4)
Supplement: Supplementary file 3 — Example of hospital-specific pneumonia feedback sheet provided to the enhanced feedback arm. (DOCX 87 kb) [file 13012_2019_868_MOESM3_ESM.docx]

**SAMPLE PNEUMONIA SPECIFIC FEEDBACK**

# correct use of amoxicillin in children with non-severe pneumonia (classified as per moh guidelines2016)

Children aged 2-59 months who present with cough or difficulty breathing are classified as non-severe pneumonia according to MoH 2016 and WHO guidelines if they have:

- No danger signs (children with danger signs have severe pneumonia)
- Lower chest indrawing OR fast breathing (Fast breathing : Respiratory rates >= 50 if aged 1-11 months and >= 40 if aged 1-4 years).

Children with TB, meningitis, severe acute malnutrition, severe malaria or those that have been re-admitted have been excluded from any analysis below.

Table 1: Target goals for implementation of new MoH pneumonia guidelines

|  | **CIN Goal** | **Hospital Performance in November 2016** | **Comments** |
| --- | --- | --- | --- |
| **Primary Goal** : prescription of high dose Amoxicillin as monotherapy for patients with non-severe pneumonia (as defined above) | **80%** | **72%** | If NO Danger signs patient can safely be managed with high dose oral amoxicillin |
| **Secondary Goal** : Classification of ALL pneumonia cases is consistent with MoH 2016 guidelines | **80%** | **82%** | **Severe pneumonia** - ANY danger sign present. **Non-severe pneumonia** - NO danger signs but has indrawing or fast breathing. |

**Continued improvements are needed to achieve the goals of better, evidence based practice. In particular please**:

- Try and ensure all non-severe pneumonia cases without danger signs are prescribed **Amoxicillin alone**
- Carefully record clinical signs of pneumonia and classify severity in accordance with guidelines

40 patients were discharged from Hospital X with admission diagnosis of pneumonia (all degrees of severity) in November. PAR forms were used in 40(100%) pneumonia admissions. Details of Hospital X's performance in November and previous months are given below.

## percentage of non-severe pneumonia cases with either indrawing or fast breathing but no danger signs prescribed high dose amoxicillin

There were 18 children who were documented to have clinical signs that indicated a diagnosis of non-severe pneumonia with either indrawing or fast breathing but no danger signs. The new MoH guidelines recommend all these children should receive treatment with high dose Amoxicillin


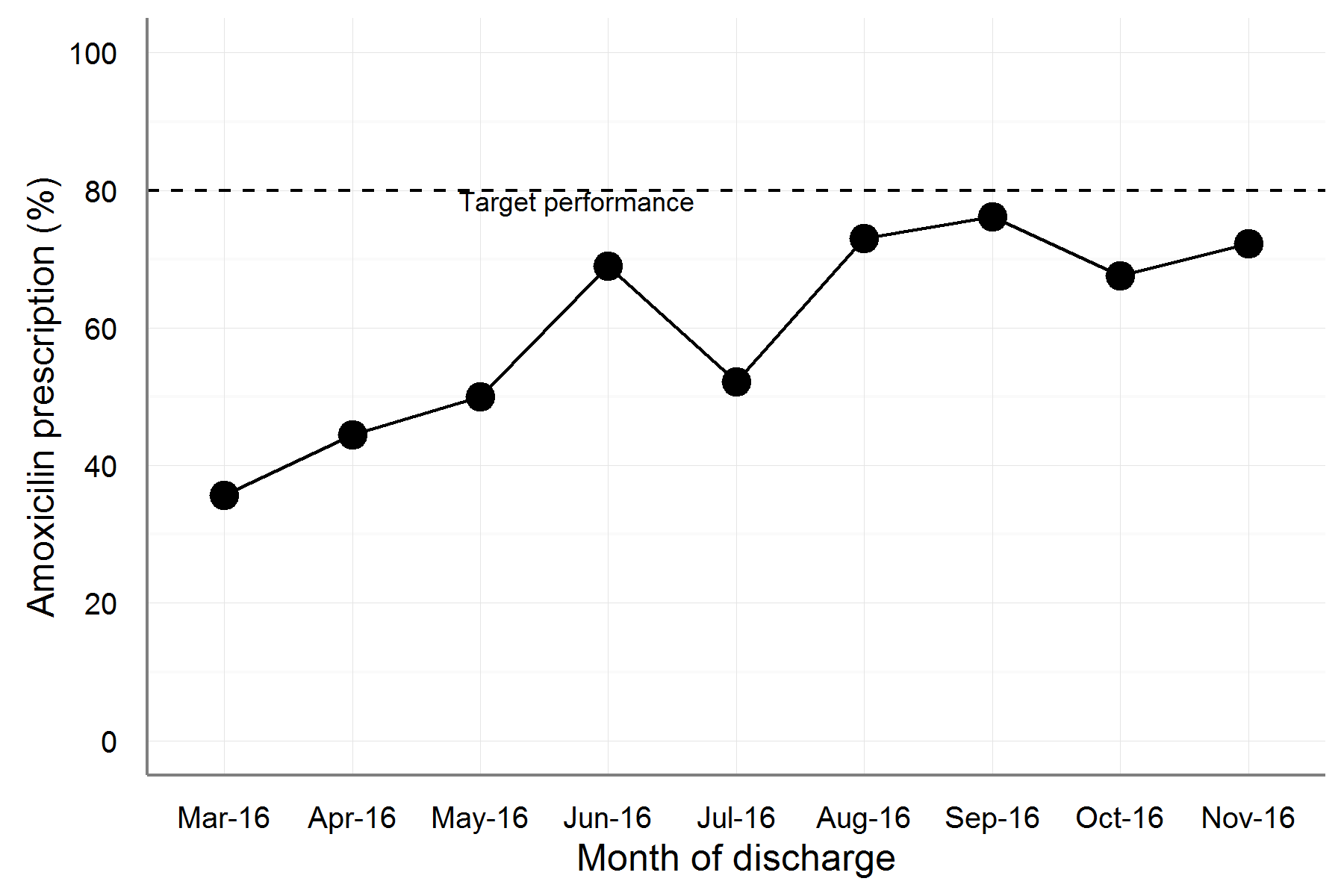


**Figure 1: Percentage of non-severe pneumonia cases prescribed amoxicillin on admission**

Table 2: Percentage of non-severe pneumonia cases prescribed amoxicillin on admission

|  | Mar-16 | Apr-16 | May-16 | Jun-16 | Jul-16 | Aug-16 | Sep-16 | Oct-16 | Nov-16 |
| --- | --- | --- | --- | --- | --- | --- | --- | --- | --- |
| Amoxicillin as a single antibiotic for children with non-severe pneumonia - Defined as cases with either Indrawing or fast breathing but no danger signs (%) | 5/14 (36%) | 8/18 (44%) | 12/24 (50%) | 20/29 (69%) | 12/23 (52%) | 27/37 (73%) | 16/21 (76%) | 23/34 (68%) | 13/18 (72%) |

# pneumonia classification

## percentage of admissions with correct classification

Ideally, the clinical signs recorded by the clinicians should be used to classify the severity of pneumonia. We use the documented clinical signs to determine severity of pneumonia as per MoH 2016 guidelines (see table below) and then compare this with the clinicians' classification.

When we check the classification based on the clinical signs you recorded we use the following rules:

| **Classification** |  |
| --- | --- |
| **Severe Pneumonia** | Presence of ANY danger sign: AVPU < A; inability to drink; grunting; cyanosis; or oxygen saturation < 90% |
| **Non-severe Pneumonia** | Indrawing or fast breathing (respiratory rates >= 50 if aged 2-11 months and>= 40 if aged 1-4 years) BUT no danger signs. |
| **Indeterminate** | Key clinical signs are not recorded so a classification based on the MoH 2016 guidelines cannot be made |

If the admitting clinician's classification of severity is not consistent with the MoH recommended classification it is regarded as not correct. Indeterminate cases are also considered incorrect.


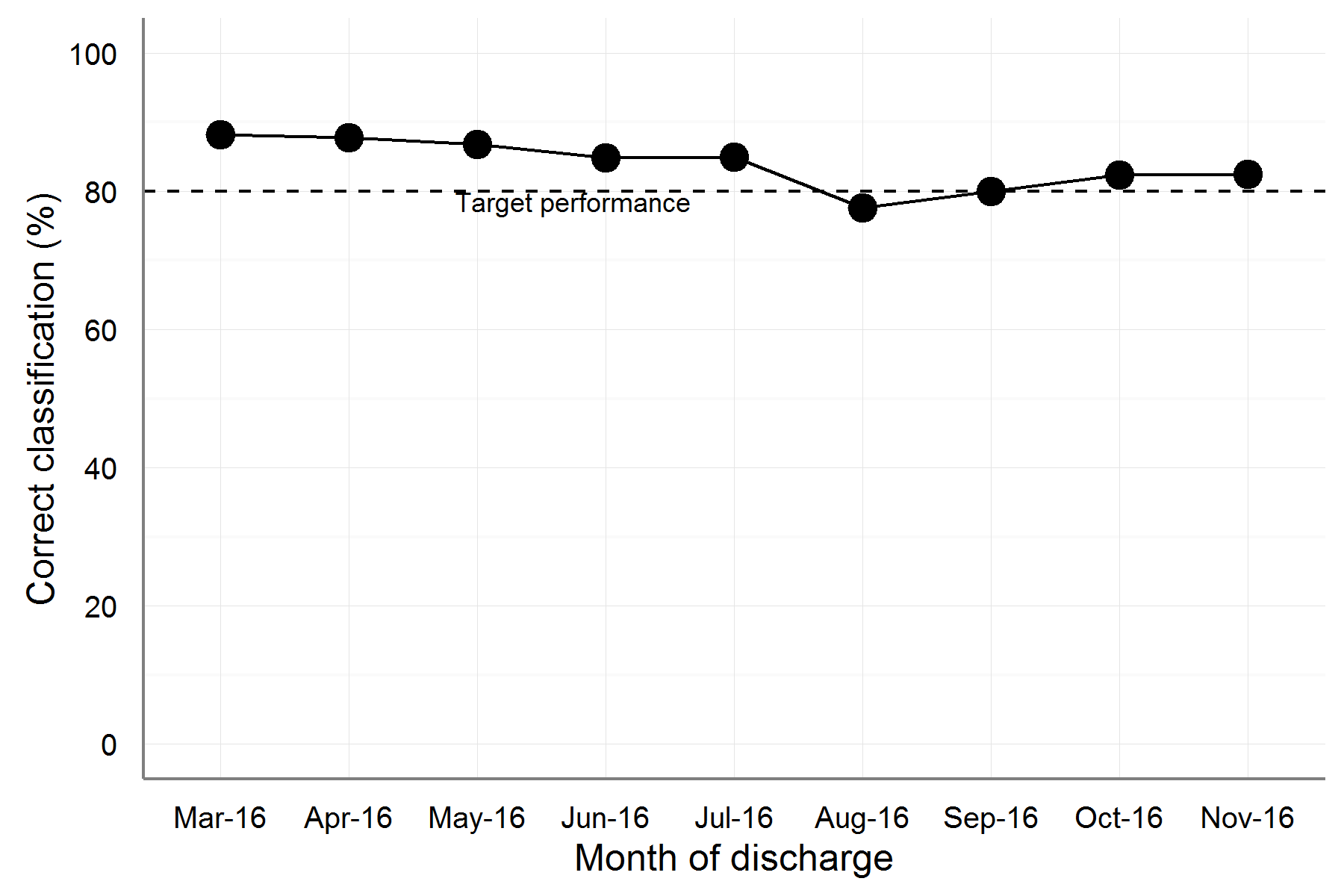


**Figure 2: Percentage of children with correct pneumonia classification**
